# TonEBP modulates the protective effect of taurine in ischemia-induced cytotoxicity in cardiomyocytes

**DOI:** 10.1038/cddis.2015.372

**Published:** 2015-12-17

**Authors:** Y J Yang, Y Y Han, K Chen, Y Zhang, X Liu, S Li, K Q Wang, J B Ge, W Liu, J Zuo

**Affiliations:** 1 Department of Cellular and Genetic Medicine, School of Basic Medical Sciences, Fudan University, Shanghai, China; 2 Department of Cardiology, Zhongshan Hospital, Fudan University, Shanghai Institute of Cardiovascular Diseases, Shanghai, China

## Abstract

Taurine, which is found at high concentration in the heart, exerts several protective actions on myocardium. Physically, the high level of taurine in heart is maintained by a taurine transporter (TauT), the expression of which is suppressed under ischemic insult. Although taurine supplementation upregulates TauT expression, elevates the intracellular taurine content and ameliorates the ischemic injury of cardiomyocytes (CMs), little is known about the regulatory mechanisms of taurine governing TauT expression under ischemia. In this study, we describe the TonE (tonicity-responsive element)/TonEBP (TonE-binding protein) pathway involved in the taurine-regulated TauT expression in ischemic CMs. Taurine inhibited the ubiquitin-dependent proteasomal degradation of TonEBP, promoted the translocation of TonEBP into the nucleus, enhanced TauT promoter activity and finally upregulated TauT expression in CMs. In addition, we observed that TonEBP had an anti-apoptotic and anti-oxidative role in CMs under ischemia. Moreover, the protective effects of taurine on myocardial ischemia were TonEBP dependent. Collectively, our findings suggest that TonEBP is a core molecule in the protective mechanism of taurine in CMs under ischemic insult.

Acute myocardial infarction (AMI) is one of the most serious cardiovascular events in humans around the world, and can be life threatening.^1, 2^ Active prevention, early diagnosis and effective treatment may be of great importance in improving the quality of life of patients with AMI. To achieve those goals, it is fundamental to clarify the mechanisms regulating cardiomyocyte (CM) death and survival during ischemic injury, and to find new effective therapies to reduce the amount of myocardial loss after a sudden coronary occlusion.^3^

Taurine, an organic acid widely distributed in animal tissues, has been reported to be involved in AMI. Under ischemic insult, the intracellular content of taurine decreases significantly in the myocardium,^4, 5, 6, 7, 8^ suggesting that taurine supplementation may be a promising therapy for AMI. Indeed, taurine has been reported to protect the heart from ischemia.^7^ These cardioprotective effects may be caused by multiple biological and physiological actions, such as anti-apoptotic and anti-oxidative effects or osmoregulatory actions and so on.^9, 10, 11, 12, 13^

Physiologically, the intracellular taurine content is maintained at a high level by the combination of endogenous biosynthesis and the membrane taurine transporter (TauT) uptake.^11, 14^ As the endogenous biosynthesis of taurine is limited in the heart, the high intracellular taurine pool mostly depends upon the uptake of taurine by TauT.^15^ The Na^+^/Cl^−^ -dependent TauT principally transports taurine from the plasma into the cells, and is a critical factor in the regulation of cellular taurine levels.^16^ However, large decreases in taurine content and TauT expression are found during ischemic insult. Importantly, pretreatment with taurine could reverse the expression of TauT,^13^ increase the cellular taurine levels and ameliorate the CMs injury. However, the mechanism of how TauT is regulated by taurine in ischemic myocardium is still elusive, and requires further studies of the molecular details.

TonEBP (tonicity-response element-binding protein), a member of the rel/NFkB/NFAT family of transcription factors, was originally identified as a transcriptional factor involved in the cellular response to hypertonic stress.^17, 18^ It has been reported that TonEBP could transcriptionally regulate the expression of several target genes responsible for the metabolism of organic osmolytes, including TauT.^10^ Furthermore, the binding site of TonEBP is located in the 5′-flanking promoter region of the TauT gene.^10^ However, the physiological role of TonEBP in the ischemic heart remains unknown, as well as its involvement in the modulation of TauT expression induced by taurine under myocardial ischemia.

In this study, we determined the essential requirement of the tonicity-responsive element (TonE)/TonEBP pathway in the upregulation of TauT gene expression induced by taurine under ischemic insult both *in vivo* and *in vitro*. We further examined the activity of TonEBP by regulating its expression, and investigated its role in the anti-apoptotic and anti-oxidative effects of taurine in ischemic CMs.

## Results

### TonEBP is a candidate molecule involved in the regulation of TauT transcription by taurine

As in previous study, we found that TauT was involved in the protective effects of taurine on CMs under ischemic insult both *in vivo* and *in vitro*, we decided to detect the mechanisms of TauT expression promotion by taurine. To determine how the TauT gene is regulated at the transcriptional level, we ran finding promoter software (http://www.ncbi.nlm.nih.gov/Class/NAWBIS/Modules/DNA/dna21b.html) using the human TauT gene promoter sequence and confirmed several DNA binding sites (*cis*-elements) for transcription factors, including TonEBP (Figure 3a). We further examined whether TonEBP, a transcriptional factor for TauT, was involved in the myocardial protective effects of taurine, which could attenuate the decrease of TauT expression under ischemic insult.

### Taurine could ameliorate the decreased TonEBP contents under ischemic insult

**Figure 1:**
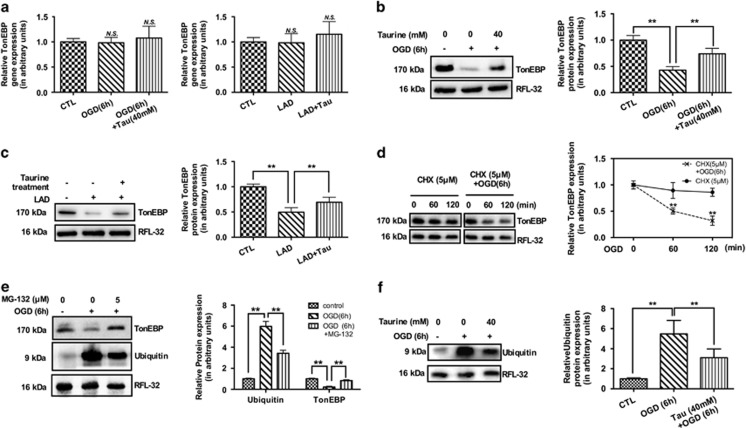
Taurine could attenuate the enhanced proteasomal degradation of TonEBP under ischemia both *in vivo* and *in vitro*. The ischemia could promote the posttranscriptional degradation of TonEBP, as the mRNA level of TonEBP did not change *in vitro* or *in vivo* (**a**, *N*=4); the protein level, however, decreased significantly (**b** and **c**, *N*=4). The supplementation of taurine could not affect the expression level of TonEBP mRNA (**a**), but could ameliorate the ischemia-induced degradation of TonEBP (**b** and **c**). The following mechanism study supported proteasomal degradation involved under ischemic insults (**d**–**f**), as the protein degraded gradually with protein synthesis inhibited by CHX (**d**, *N*=3), and the proteasome inhibitor, MG-132, could inhibit such degradation (**e**, *N*=3). Furthermore, taurine could inhibit the activation of ubiquitin-proteasomal degradation of TonEBP (**f**, *N*=3). Therefore, we confirm that taurine could attenuate the posttranscriptional degradation of TonEBP by inhibiting the ubiquitin-proteasomal pathway. Staining for RFL-32 was performed to indicate approximate equal loading of samples. (Data were expressed as mean±S.E.M. **P<0.01; CHX, cycloheximide; CTL, control; NS, no significance; LAD, left anterior descending coronary artery ligation; OGD, oxygen glucose deprivation; Tau, taurine)

To determine the expression of TonEBP messenger RNA (mRNA) and protein, quantitative real-time PCR and western blotting were performed. Under ischemic insult, no changes in TonEBP mRNA were observed in human CMs (HCMs) or in rats' cardiac tissues (Figure 1a). Similarly, when treated with taurine, the TonEBP mRNA levels showed no significant changes (Figure 1a). However, the TonEBP protein decreased significantly in ischemic models (Figures 1b and c), and was ameliorated by taurine supplementation (Figures 1b and c).

### Taurine could attenuate the enhanced proteasomal degradation of TonEBP

The TonEBP mRNA did not change with the reduction of TonEBP protein under ischemic insult, leading us to detect the posttranslational regulation of TonEBP. To ascertain whether the protein stability was affected in myocardial ischemia, the protein synthesis inhibitor cycloheximide (CHX, 5 μM) was administered. In the conditions of protein synthesis inhibition, the turnover of TonEBP protein was accelerated in HCMs under ischemia, indicating increased proteolysis of TonEBP in ischemia (Figure 1d). Therefore, the involvement of proteasome-mediated proteolysis in the reduction of TonEBP protein under ischemia was investigated. Treatment with the proteasome inhibitor MG-132 (5 μM) significantly prevented the downregulation of TonEBP (Figure 1e), suggesting the involvement of proteasome activation in the enhanced degradation of TonEBP under ischemia. When treated with taurine, the enhanced expression of ubiquitin was ameliorated (Figure 1f), supporting the conclusion that taurine could attenuate the enhanced ubiquitin-proteasomal degradation of TonEBP in myocardial ischemia.

### Taurine could promote the translocation of TonEBP to the nucleus

**Figure 2:**
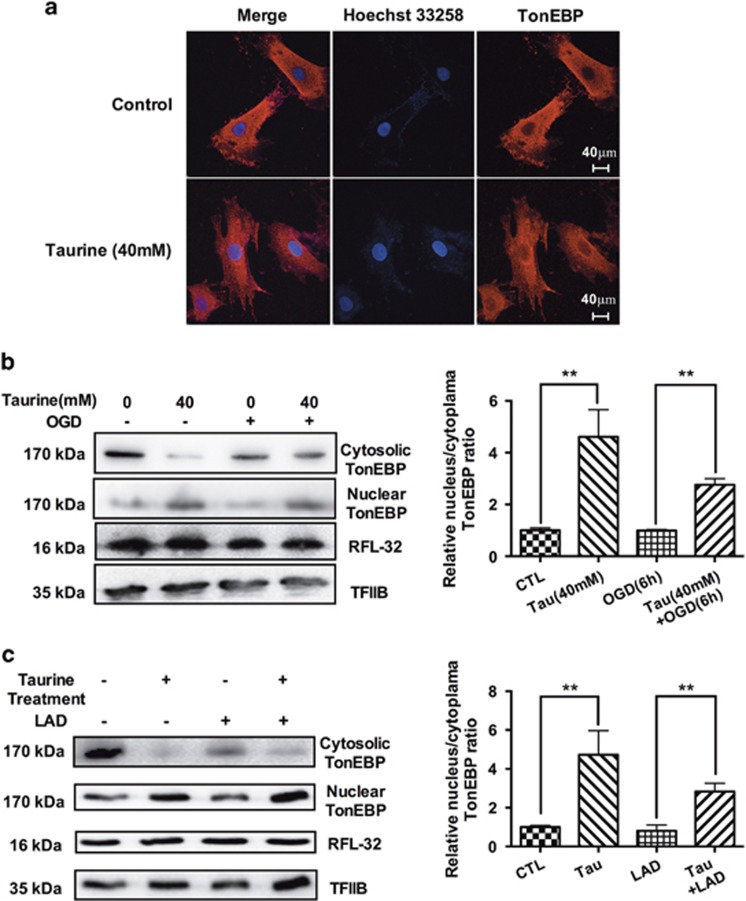
Taurine could promote the translocation of TonEBP to the nucleus. In our study, taurine could promote the nuclear localization of TonEBP as detected by immunofluorescence (**a**), decreasing the cytosolic TonEBP and increasing the nuclear TonEBP both *in vivo* and *in vitro* (**b** and **c**, *N*=3). The results were similar in the normal condition and under ischemia. Staining for RFL-32 was performed to indicate approximate equal loading of samples. Staining for TFIIB was performed to indicate approximate equal loading of nuclear samples. (Data were expressed as mean±S.E.M. **P<0.01; LAD, left anterior descending coronary artery ligation; OGD, oxygen glucose deprivation)

In addition to the mRNA and protein expression levels of TonEBP, we also detected the distribution of TonEBP in CMs. We observed that the treatment with taurine could decrease cytosolic TonEBP, increase the nuclear TonEBP and promote the translocation of TonEBP to the nucleus (Figure 2a). This translocation was also present under conditions of myocardial ischemia both *in vivo* and *in vitro* (Figures 2b and c).

### Taurine could promote TauT transcription via TonEBP-TonE occupancy in the TauT promoter region

**Figure 3:**
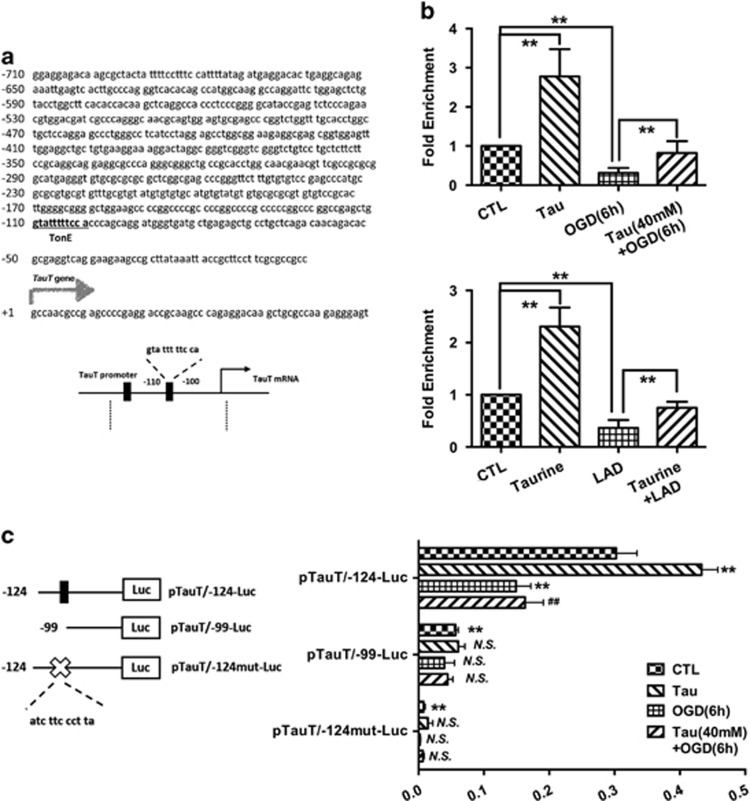
Taurine could promote the transcriptional activity of TauT by TonEBP-TonE occupancy in the TauT promoter region. (**a**) Analysis of TauT promoter showed a conserved TonE-binding sites at −110 bases and −109 bases from the transcription start site. (**b**) In the ChIP assay, the TonEBP-TonE occupancy decreased significantly under ischemic insults, and taurine could increase the binding of TonEBP with TonE significantly both *in vivo* and *in vitro* (*N*=6). (**c**) The proximal site of TauT promoter region (−99 to −124 bp) functions as an efficient *cis*-element of TonEBP, in the ischemia and taurine-modulated regulation of TauT (*N*=4). The luciferase activities in cells transfected with pTauT/-124-Luc decreased significantly under ischemia, whereas taurine strongly attenuated such reduction. In the cells transfected by the pTauT/-99-Luc or pTauT/-124mut-Luc reporter, the luciferase activities decreased to an extremely low level. The luciferase activities (data were expressed as mean±S.E.M. **P<0.01; ^##^*P*<0.01 *versus* the OGD group in **c**; NS, no significance *versus* the control group; CTL, control group)

To detect whether TonEBP-TonE occupancy could be identified within the TauT promoter region, a chromatin immunoprecipitation (ChIP) assay was performed. In ischemic HCMs and the CMs of rats, the TonEBP-TonE occupancy in the TauT promoter decreased significantly (Figure 3b). When treated with taurine, the TonEBP-TonE occupancy increased significantly (Figure 3b).

To determine which site functions as an important *cis*-element for the promoter activity of TauT, a series of deletion reporter constructs (pTauT/-124-Luc and pTauT/-99-Luc) and a mutant reporter construct (pTauT/-124mut-Luc) were constructed and transiently transfected into HCMs. Between the two deletion constructs, pTauT/-99-Luc with a deletion of the proximal TonE region (TonEBP-binding site) markedly decreased luciferase activity compared with the pTauT/-124-Luc reporter construct (Figure 3c). In addition, the decreased promoter activity of pTauT/-124-Luc was induced by ischemic insult, whereas taurine strongly attenuated the reduction in reporter activity (Figure 3c). Still, in the cells transfected with the pTauT/-99-Luc reporter, no significant regulation of TauT activity could be found in response to ischemia or taurine treatment (Figure 3c). Furthermore, the reporter activity of the mutated TonEBP-binding region, pTauT/-124mut-Luc, was not affected by ischemia or taurine. The luciferase reporter assay supported the proximal site of the TauT promoter region (−99 to −124 bp) as an efficient *cis*-element of TonEBP in the ischemia and taurine-modulated regulation of TauT (Figure 3c).

### TonEBP could regulate TauT expression and taurine content in HCMs

To confirm whether restoring cardiac TonEBP levels could confer protection against ischemia, the TonEBP gene was cloned into an expression plasmid and expressed in HCMs. The expression of TonEBP was confirmed by real-time PCR and western blotting (Supplementary Figure 1). HCMs with TonEBP overexpression or knockdown were then subjected to oxygen glucose deprivation (OGD).

**Figure 4:**
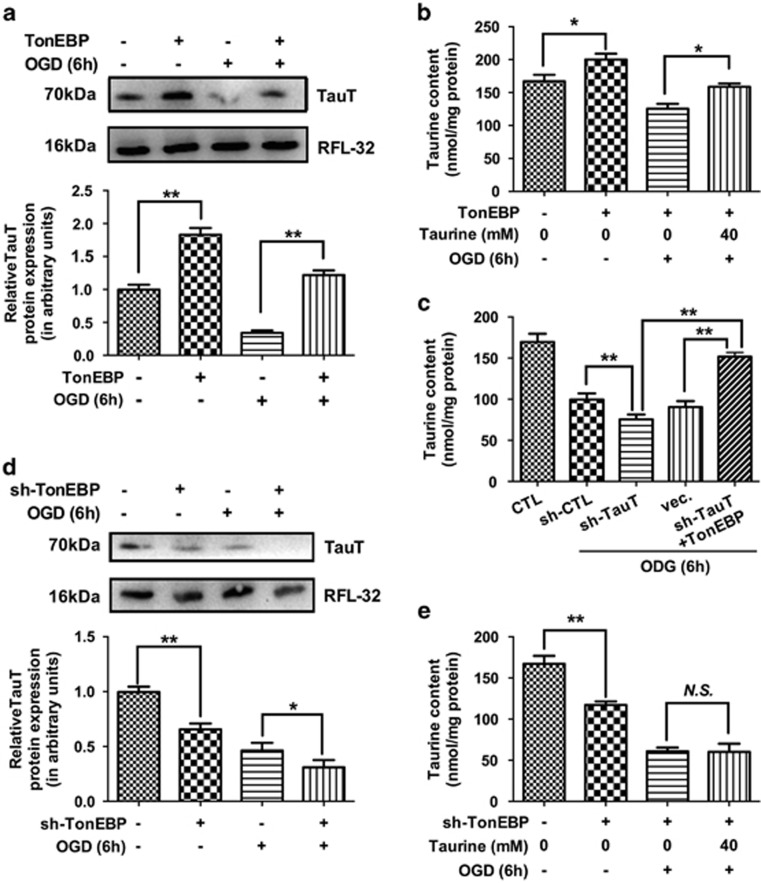
TonEBP could regulate TauT expression and taurine content in HCMs. In our study, TonEBP overexpression could upregulate TauT expression (**a**, *N*=3), increase the intracellular taurine contents (**b**, *N*=4) with or without ischemic insults. The similar results could also be confirmed in the condition of TauT knockdown (**c**, *N*=4). TonEBP knockdown could decrease TauT expression (**d**, *N*=3), further decrease the taurine levels, and even exogenous taurine supplementation could not upregulate taurine content (**e**, *N*=4). Staining for RFL-32 was performed to indicate approximate equal loading of samples. (Data were expressed as mean±S.E.M. *P<0.05; **P<0.01; NS, no significance; OGD, oxygen glucose deprivation)

Next, we detected the effects of TonEBP on TauT expression and intracellular taurine content in HCMs. In our study, TonEBP overexpression could upregulate TauT expression (Figure 4a) and increase the intracellular taurine contents (Figure 4b). Similar results were observed for the ischemic condition and in HCMs with TauT knockdown (Figure 4c). Moreover, TonEBP knockdown decreased TauT expression (Figure 4d) and further accelerated the decreased taurine levels. Even exogenous taurine supplementation could not upregulate the taurine content in HCMs with TonEBP knockdown (Figure 4e).

### TonEBP exhibited anti-apoptotic and anti-oxidative effects in HCMs under ischemic insult

**Figure 5:**
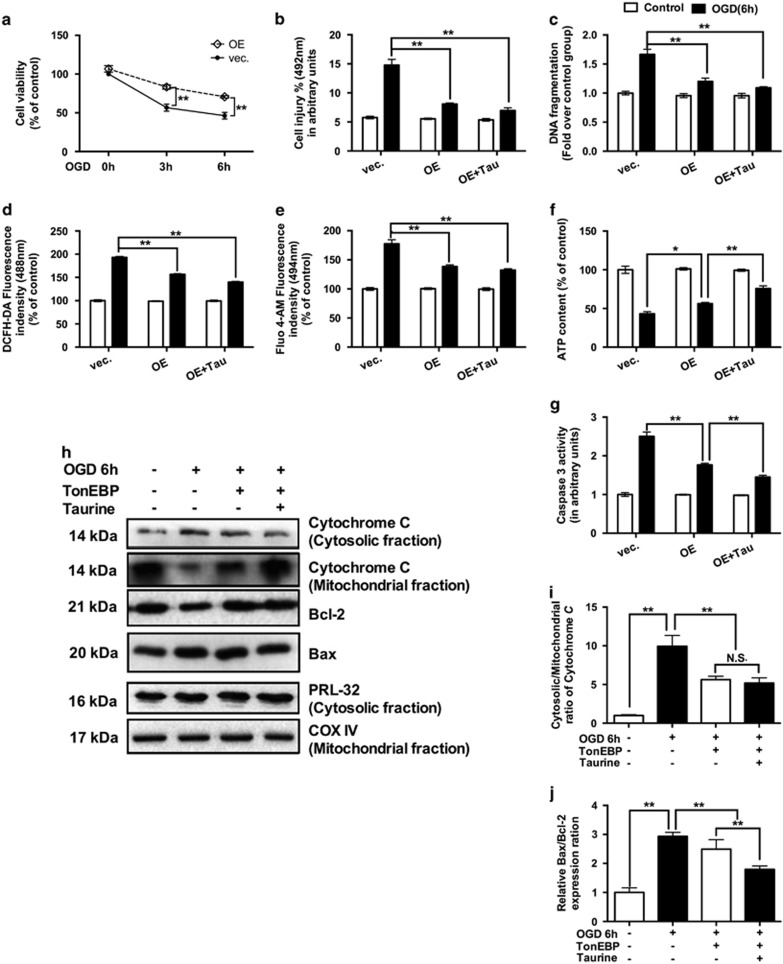
TonEBP exhibited anti-apoptotic and anti-oxidative effects in HCMs under ischemia. TonEBP overexpression significantly increased the ratio of cell viability (**a**, *N*=3), decreased cell injury (**b**, *N*=3) and attenuated DNA fragmentation (**c**, *N*=3) in HCMs under the condition of OGD. Similarly, the TonEBP overexpression could attenuate the increased ROS (**d**, *N*=3) and calcium content (**e**, *N*=3) and ameliorate the depletion of ATP content (**f**, *N*=3) in OGD HCMs. Also, TonEBP overexpression could significantly reduce caspase-3 activity (**g**, *N*=3), inhibit the release of cytochrome c (**h** and **i**, *N*=3) and decrease the ratio of Bax/Bcl-2 (**h**, **j**, *N*=6) in HCMs under ischemic insults. Staining for RFL-32 was performed to indicate approximate equal loading of samples. Staining for COX IV was performed to indicate approximate equal loading of mitochondrial samples. (Data were expressed as mean±S.E.M. *P<0.05; **P<0.01; NS, no significance; OE, overexpression; OGD, oxygen glucose deprivation; Tau, taurine; Vec, vector)

**Figure 6:**
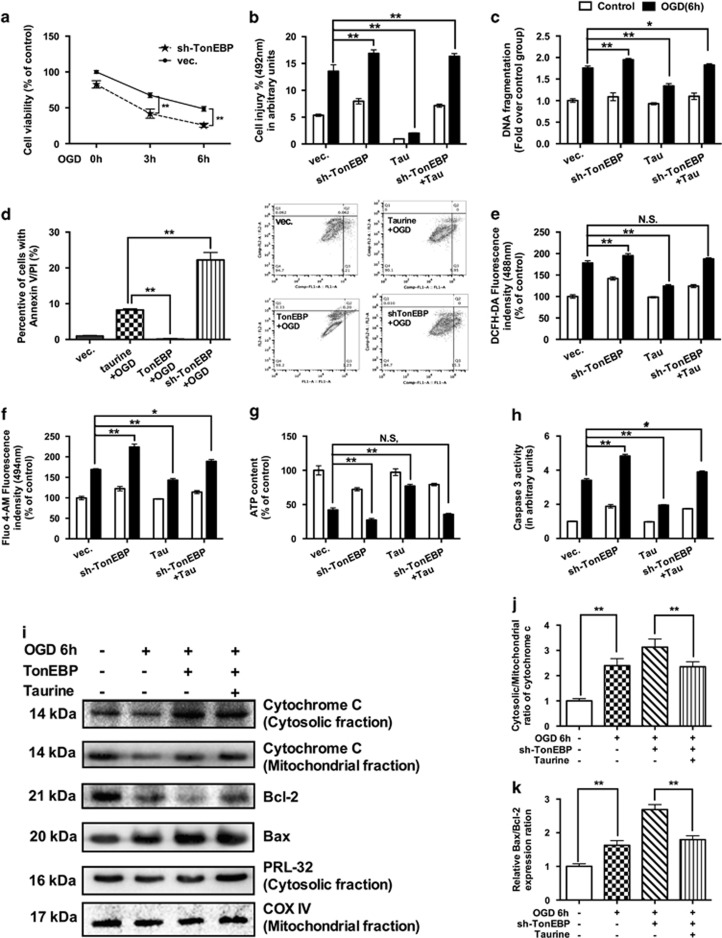
The protective effects of taurine on ischemic HCMs are TonEBP dependent. The knockdown of TonEBP decreased the ratio of cell viability (**a**, *N*=3), increased the LDH release level (**b**, *N*=3), increased the DNA fragmentation (**c**, *N*=3) and Annexin V-FITC and PI staining (**d**, *N*=3). Taurine supplementation could decrease the ratio of cell death and apoptosis (**d**, *N*=3). In the oxidative stress tests, TonEBP knockdown could exacerbate ROS production (**e**, *N*=3), calcium overload (**f**, *N*=3) and ATP depletion (**g**, *N*=3), associated with increased levels of caspase-3 (**h**, *N*=3). Similarly, both the Bax/Bcl-2 ratio (**i** and **j**, *N*=3) and cytochrome c release (**i**, **k**, *N*=6) were enhanced in TonEBP knockdown groups. With TonEBP knockdown, the further treatment of taurine no longer reverse the myocardial injury caused by OGD (**b**–**h**). (Data were expressed as mean±S.E.M. *P<0.05; **P<0.01; NS, no significance; OGD, oxygen glucose deprivation; Tau, taurine; Vec, vector)

TonEBP overexpression significantly increased the ratio of cell viability (Figure 5a), decreased cell injury as detected by lactate dehydrogenase (LDH) assays (Figure 5b) and attenuated DNA fragmentation (Figure 5c) in HCMs under the condition of OGD. The protection elicited by TonEBP overexpression was also confirmed by a reduction in apoptosis in cells stained by Annexin V-FITC and propidium iodide (PI) staining (Figure 6d).

In the anti-oxidative stress tests, the overexpressed TonEBP could attenuate the increased ROS (Figure 5d) and calcium content (Figure 5e), and ameliorate the depletion of ATP content (Figure 5f) in HCMs under OGD. In addition, TonEBP overexpression could significantly reduce caspase-3 activity in HCMs under the condition of OGD (Figure 5g). Furthermore, overexpressing TonEBP could also inhibit the ratio of Bax/Bcl-2 and the release of cytochrome c in HCMs under ischemic insults (Figures 5h and j). Similarly, such results were observed for the positive control of hydrogen peroxide (Supplementary Figures 2A and B).

### The protective effects of taurine on ischemic HCMs are TonEBP dependent

As taurine could attenuate the degradation of TonEBP, protecting the HCMs from ischemic insults, we evaluated the involvement of TonEBP in the myocardial protective effects induced by taurine. In this study, reducing TonEBP expression by the knockdown of TonEBP was associated with decreased cell viability (Figure 6a) and increased LDH release level (Figure 6b) after OGD. With TonEBP knockdown, the extent of apoptosis in CMs was elevated after 6 h of OGD, as indicated by significant increases in DNA fragmentation (Figure 6c) and Annexin V-FITC and PI staining (Figure 6d). Furthermore, the supplementation of taurine could not ameliorate those apoptotic effects induced by OGD, under conditions of TonEBP knockdown (Figures 6b and c).

In the anti-oxidative stress tests, our study indicated that TonEBP knockdown could exacerbate oxidative stress, including ROS production (Figure 6e), calcium overload (Figure 6f) and ATP depletion (Figure 6g), associated with increased levels of caspase-3 (Figure 6h). Similarly, both the Bax/Bcl-2 ratio and cytochrome c release were enhanced in TonEBP knockdown groups (Figures 6i and k). With TonEBP knockdown, additional treatment with taurine no longer reversed the myocardial injury caused by OGD (Figures 6b and h). All of these data supported conclusion that the protective effects of taurine on CMs ischemia are TonEBP dependent.

## Discussion

In this study, we demonstrated that taurine inhibited proteasomal degradation of TonEBP under ischemia and promoted the translocation of TonEBP to the nucleus. When translocated to the nucleus, TonEBP occupied the promoter region of the TauT gene and induced TauT expression in CMs. Moreover, we correlated the protective effects of TonEBP with its ability to upregulate TauT expression, increase the content of taurine and thereby prevent the cell death of CMs by anti-apoptotic and anti-oxidative effects. Meanwhile, our study confirmed that the protective effects of taurine against ischemic insult were TonEBP dependent. Collectively, our observations suggested that the TonEBP/TauT/taurine pathway is an important target in myocardial infarction.

Taurine has been implicated in the pathogenesis, diagnosis and treatment of heart disease in the past few decades.^19, 20, 21^ Alterations and abnormal metabolism of several free amino acids, including taurine, were found in myocardium and plasma of patients with myocardial infarction.^19^ Taurine in the myocardium was reported to decrease during the ischemic process in several studies, suggesting that taurine is a possible biomarker in the diagnosis of myocardial ischemia.^20^ In addition, taurine content was decreased in many other heart diseases, such as hypertension and cardiac hypertrophy,^21^ implying that taurine may have a role in myocardial protection.^20^ However, the protective effects and the mechanisms of taurine on heart disease remain to be elucidated. Our previous study confirmed that taurine contents were lower in CMs under ischemic insult *in vivo* and *in vitro* and that exogenous taurine could prevent ischemia-induced cell apoptosis via attenuated oxidative stress.^12, 13^ In that process, TauT expression decreased in myocardial ischemia, and the supplementation of taurine could promote TauT expression, increasing exogenous taurine uptake.^13^ However, the mechanisms of TauT expression affected by ischemia and taurine treatment have not been illuminated.

The regulation of TauT has been observed previously in various organs such as kidney, liver, heart and brain.^10, 18, 22, 23^ In those studies, TonEBP has been suggested to be a possible key molecule involved in the transcription of a number of target genes, including TauT.^24, 25^ Interestingly, we found that the level of TonEBP in myocardium decreased when TauT decreased in ischemic rats. Therefore, we hypothesized that TonEBP is involved in the expression of TauT induced by taurine under ischemic insult.

TonEBP is abundantly expressed in the brain, heart, liver, and activated T cells,^18^ and the activity of TonEBP may be regulated at multiple levels, including its abundance, nuclear localization and phosphorylation.^26^ In this study, we first evaluated the abundance of TonEBP. In ischemia, the mRNA level of TonEBP was not affected, but an increased proteasome-dependent degradation of TonEBP was reported in CMs. Furthermore, taurine could inhibit the expression of ubiquitin and attenuate the proteasomal degradation of TonEBP in ischemia, supporting our hypothesis that TonEBP was involved in the protection effects of taurine. In previous studies, the degradation of TonEBP was rarely discussed. However, the proteasome was reported to be involved in some nuclear translocation of TonEBP,^25^ and the proteasome inhibitor MG-132 could decrease the ratio in hypertonic cells in a dose-dependent manner.^24^ Therefore, the exact role the proteasome in the degradation and nucleocytoplasmic trafficking of TonEBP is still elusive, and it should be further studied.

In addition to inhibiting TonEBP degradation, taurine further enhanced the translocation of TonEBP into the nucleus in our study. This finding could be supported by the fact that the activity of TonEBP was regulated mainly at the level of nucleocytoplasmic distribution,^27^ and the activated TonEBP could further enhance the mRNA expression of the downstream genes.^28^ When translocated into the nucleus, the detailed mechanisms of TonEBP on the transcriptional activity of TauT were further explored.

We detected the effects of TonEBP on the transcriptional activity of TauT, and identified an ischemia-related TonE consensus motif from −110 bp to −100 bp of the TauT promoter region as an efficient *cis*-element for TonEBP. ChIP analysis confirmed that TonEBP constitutively occupied the TauT promoter region and that the TonEBP-TonE occupancy in the TauT promoter region was decreased in ischemic myocardium. In addition, luciferase assays demonstrated that taurine incubation promoted TonEBP-TonE occupancy. In CMs transfected with pTauT/-124-Luc, luciferase transcription was activated after exposure to taurine with or without ischemia. On the other hand, when the CMs were transfected with pTauT/-99-Luc, which lacks a TonE site, taurine did not influence luciferase expression. Furthermore, mutation of the TonE sequence abolished the induction of promoter activation in response to taurine, as observed in cells transfected with pTauT/-124mut-Luc. Thus, this site is essential and sufficient for the activation of TauT promoter by taurine in the presence or absence of ischemic conditions. Collectively, these data suggest that the translocated TonEBP in the nucleus could bind with the TonE consensus motif and activate the TauT transcription activity. Similar phenomenon could also be found in the condition of hypertonicity and heat-shock stresses, respectively,^29, 30, 31^ supporting the finding of this study.

In addition to regulating the transcriptional activity of TauT, we also explored the role of TonEBP in alleviating oxidative stress and apoptosis during ischemia. It was reported that reactive oxygen species (ROS), a component of oxidative stress, contributed to transactivation of TonEBP, and the activation of TonEBP was associated with suppression of ROS formation.^32^ In our study, the knockdown of TonEBP led to ROS accumulation during ischemia, whereas TonEBP overexpression mitigated the oxidative effect of ischemia and H_2_O_2_, supporting TonEBP an anti-oxidative stress molecule. Moreover, TonEBP was reported to have a role in the apoptosis of retinal neurons;^33^ however, the mechanism of TonEBP in apoptosis was not fully discussed. In our study, inhibited TonEBP expression exacerbated cell death and apoptosis during ischemia, whereas overexpression of TonEBP increased survival of CMs during OGD, intimating the involvement of endogenous TonEBP in the regulation of survival/death during OGD. Our study supported that TonEBP is a key molecule in anti-oxidative stress and anti-apoptosis, and such results could be supported by previous studies in kidney and liver cells, where TonEBP promoted cell survival and inhibited apoptotic cell death in response to hypertonic conditions.^10, 34^ Furthermore, in the condition of TonEBP knockdown, the protective effects of taurine were blocked. Therefore, the anti-oxidative and anti-apoptotic effects of taurine are TonEBP dependent. All of these results show a central role for TonEBP in the protective effects of taurine under ischemia.

### Conclusions

In this study, we demonstrated a central role of the TonEBP/TauT pathway in the cardioprotective effects of taurine under AMI. Our study may promote the understanding of how TauT is modulated after treatment with taurine, and offer more evidence for further clinical applications. More work is needed to explore the detailed mechanism of the regulation of TauT expression. Therapies focusing on those pathways may have a bright future.

## Materials and Methods

### Animals and procedures

The animals were pathogen-free, adult male Sprague-Dawley (SD) rats, weighing 250–300 g (Shanghai Institute of Materia Medica, Chinese Academy of Sciences, Shanghai, China). The rats were randomly assigned into four groups (*n*=6 per group): (i) control group (untreated sham-operated group), (ii) taurine-treated control group (sham-operated control group treated with taurine), (iii) left anterior descending coronary artery ligation (LAD) group and (iv) taurine-treated LAD group. Before modeling, all rats received an intraperitoneal injection of 1 ml physiological saline or 100 mg/kg/day of taurine (Sigma-Aldrich, St Louis, MO, USA) for 3 consecutive days. After anesthetization by intraperitoneal injections of ketamine (100 mg/kg), and shaving on the chest, the rats were then placed in a supine position and intubated with positive-pressure ventilation (180 ml/min) with room air using a SAR-830/A Small Animal Ventilator (CWE, Inc., Weston, WI, USA). Under sterile conditions, the heart was exposed via a left thoracotomy at the level of the fifth intercostal space. LAD was created by left coronary artery ligation 2 mm below the left atrium with a 6.0 Prolene suture. Regional myocardial ischemia was confirmed through the observation of a rapid discoloration over the anterior surface of the left ventricle together with the development of akinesia and dilation over the area at risk. The sham-operated control rats underwent thoracotomy without left coronary artery ligation. In each group, the animals were killed 30 min after modeling. The experimental protocol was approved by the Shanghai Medical Experimental Animal Care and Use Committee.

### Cell culture and treatment

The HCMs were kindly provided by Professor Wang Keqiang of the Shanghai Institute of Cardiovascular Diseases, and were maintained at 37 °C with 5% carbon dioxide (CO_2_) in air atmosphere in Dulbecco's modified Eagle's medium (DMEM) (Gibco, Carlsbad, CA, USA) supplemented with 10% (volume/volume) heat-inactivated fetal bovine serum (Gibco) and antibiotics (100 U/ml penicillin and 100 mg/ml streptomycin) (Gibco). For OGD conditions, the cells were cultured in no-serum, no-glucose DMEM (Gibco) at 37 °C with 1% oxygen and 5% CO_2_ in a hypoxic incubator (Ruskinn Sci-tive All inclusive, Ruskinn Technology, Bridgend, UK).

### RNA isolation and real-time polymerase chain reaction (PCR)

Total RNA was isolated from rats and HCMs using Trizol reagent (Life Technologies, Carlsbad, CA, USA) according to the manufacturer's instructions. After quantification and determination of the quality of total RNA, the first-strand cDNA was synthesized with a ReverAid first-strand cDNA synthesis kit (Thermo Scientific, Rockford, IL, USA). The mRNA level was determined by real-time PCR using SYBR Premix Ex Taq II (TaKaRa, Tokyo, Japan). PCR amplification cycles were programmed at 95 °C for 30 s, followed by 40 cycles of 95 °C for 30 s, 60 °C for 30 s and 72 °C for 40 s. *β*-Actin was used as an endogenous control. The relative expression of genes was then calculated. The following primers were used; for TauT: 5′-AGCTAGCGGTGTATGCCTTT-3′ and 5′-TCACAGCCCAGCTGTACTTC-3′ for TonEBP: 5′-CCTGCAGGTAACTGGACGA-3′ and 5′-TAGGATCAAGGCCGACTTCT-3′ and for *β*-actin: 5′-AGATGAAGCTCTCCCTGGTG-3′ and 5′-CACGTCCTCCTTCTTGTCCT-3′. All the primers having been tested for optimal specificity and efficiency with the thermal program used.

### Immunoblotting

Cardiac tissues or HCMs were harvested into 1ml RIPA lysis buffer (Thermo Pierce, Rockford, IL, USA) supplemented with complete protease inhibitor cocktail (Roche Applied Science, South San Francisco, CA, USA). The lysates were centrifuged at 13 500 × *g* for 30 min at 4 °C and the total protein content of the supernatant fraction was measured by using the BCA protein assay kit (Thermo Pierce). Nuclear and cytosolic protein fractions were prepared by NE-PER nuclear and cytoplasmic extraction reagents (Thermo Pierce) according to the manufacturer's protocol. Equal amounts of protein (40 mg per lane) were separated by sodium dodecyl sulfate polyacrylamide gel electrophoresis and transferred to a 0.45 mm PVDF membrane (Millipore, Billerica, MA, USA). The membrane was blocked with 5% skimmed milk in TBS-T, and incubated with affinity-purified antibodies against RPL-32 (1 : 3000, Abcam, Cambridge, MA, USA), TauT (1 : 1000, Abcam), TonEBP (1 : 1000, Abcam), ubiquitin (1 : 1000, Cell Signaling Technology, Danvers, MA, USA), TFIIB (1 : 1000, Cell Signaling Technology), cytochrome c (1 : 1000, Cell Signaling Technology), Bcl-2 (1 : 1000, Cell Signaling Technology), Bax (1 : 1000, Cell Signaling Technology) and COX IV (1 : 2000, Cell Signaling Technology), and incubated with appropriate horseradish peroxidase (HRP)-conjugated secondary antibodies (1 : 6000, Abcam). Finally, proteins were detected using the Immobilon western chemiluminescent HRP substrate (Bio-Rad, Hercules, CA, USA) and images were captured with the Gel Doc XR System (Bio-Rad Laboratories, Hercules, CA, USA).

### Protein degradation assay

TonEBP protein degradation under ischemic insult was assayed by CHX-chase analysis (Sigma-Aldrich). After pretreatment with CHX (5 μM), the protein abundance of TonEBP was determined via western blot.

### Ubiquitin-proteasomal assay

HCMs were treated with or without proteasome inhibitor MG-132 (5 μM, Sigma-Aldrich) for 2 h. Total protein was harvested and lysed in RIPA lysis buffer. The protein level of TonEBP and ubiquitin were determined by western blotting.

### Immunofluorescence studies

HCMs were stained with polyclonal antibodies to TonEBP (1 : 200, Abcam), followed by incubation with labeled secondary antibodies (goat anti-rabbit IgG(H+L), Secondary Antibody, Alexa fluor 594 Conjudate, 1 : 3000, Life Technologies), as described earlier.^12^ After mounting with Hoechest 33258 (Life Technologies), the cells were visualized with a scanning confocal microscope (Nikon ECLIPSE Ti, Nikon, Japan).

### ChIP assay

The binding of TonEBP to DNA was evaluated using a ChIP assay kit (Promega, Madison, WI, USA) according to the manufacturer's instructions. HCMs were cross-linked with 1% formaldehyde for 10 min at room temperature; the lysates were sonicated six times for 40 s each time using a Bioruptor (Diagenode, Liege, Belgium). After centrifugation, the supernatant was diluted in ChIP dilution buffer and then incubated overnight at 4 °C with the TonEBP primary antibody. The eluted DNA was quantified by real-time PCR and normalized to the IgG control.

### Luciferase reporter assay

In this part, we obtained the human TauT promoter-driven luciferase reporter plasmid pGL3 (Promega) by using a 1100-bp fragment of the TauT promoter region DNA as the templates for PCR. The PCR fragment was then cloned into the pGL3-Basic or SV40/pGL3 control vector (Promega). The TauT promoter was amplified by PCR using Q5 High-Fidelity DNA Polymerases (New England Biolabs, Ipswich, MA, USA) (forward, 5′-GGGGTACCTTACTGAAGGTCACACAGC-3′ reverse, 5′-AAGATCTTGGCACGGGAGTTCA-3′) from genomic HCMs and sequenced.

The TauT deletion constructs of pGL3/TauT (−124 to +46 bp and −99 to +46 bp) and the TauT mutation construct of pGL3/TauT/-124-Luc were generated by PCR.

The plasmid was transfected into HCMs using HilyMax (Dojindo, Tokyo, Japan). Transfection was carried out for 24 h. The cells were washed twice with PBS and incubated with taurine-containing medium for 24 h before harvesting. pGL-control and the empty pGL-Basic vectors were used as positive and negative controls. The cells were harvested 24 h after transfection and lysed in 200 μl of reporter lysis buffer (Promega). A luciferase assay was performed using a luciferase assay kit (Promega), and the activity was measured with an Optocomp 1 luminometer (MGM Instruments, Inc., Hamden, CT, USA). The promoter activity of each construct is represented by relative light output normalized to the pRL-CMV control.

### Transient transfection

To achieve TonEBP overexpression, a cDNA encode with TonEBP was amplified using recursive PCR strategy (TonEBP primers: forward: 5′-ATGCCCTCGGACTTCATCTC-3′ reverse: 5′-GAACAACTTGACTGGCTCCTTTTAA-3′). Then, TonEBP cDNA was subcloned into the pLenti6 vector using HilyMax Liposomal Transfection Reagents (Dojindo) according to the manufacturer's instructions. After sequencing, the overexpression of TonEBP was confirmed by western blotting using a specific anti-TonEBP antibody (Abcam).

### Treatment of HCMs with shRNA against TauT and TonEBP

HCMs were transfected with either TauT shRNA(h) plasmid (catalog no. sc-61648-SH, Santa Cruz, CA, USA) to block the expression of TauT or with TonEBP shRNA(h) plasmid (catalog no. sc-43968-SH, Santa Cruz) to block the expression of TonEBP using HilyMax Transfection Reagent according to the manufacturer's protocol. Control shRNA Plasmid-A (catalog no. sc-108060, Santa Cruz) was used as a negative control.

### Measurement of intracellular taurine level

To measure the taurine content in the CMs, the medium was removed and replaced with standard incubation buffer (mM: KCl 3, NaCl 125, KH_2_PO_4_ 12, MgSO_4_ 1.2, CaCl_2_ 1.3, NaHCO_3_ 5, HEPES 20, pH 7.4 and 0.2% bovine serum albumin). After that, the intracellular taurine level was measured by reversed-phase high-performance liquid chromatography. Approximately 1 × 10^6^ CMs were dissolved in 0.4 m perchloric acid (HClO_4_) (Sigma-Aldrich) and stored at −80 °C until use. The supernatants were purified in a dual-bed ion-exchange column, eluted with 2 ml water and then lyophilized. The samples were dissolved in 100 ml ultrapure water with a Nova-Pak C18 column (Bio-Rad Laboratories). Glutamine was used as an authentic standard. The standard curve of taurine content was linear and the recovery of taurine was 90%. The value of taurine was expressed as nanomoles per milligram protein.

### Assessment of cell viability

Cell viability was measured by the CCK-8 test (Dojindo). Briefly, cells were plated in 96-well plates at a density of 4 × 10^4^ cells per well. After being pretreated with 40 mM taurine for 24 h, the cells were maintained under ischemic condition for 3 or 6 h. Then, 10 ml of CCK-8 reagent was added into each well and the plates were incubated for another 1 h until the media turned yellow. The optical density was determined at 450 nm using a Multiskan MK3 microplate reader (Thermo Electron, Rockford, IL, USA).

### LDH activity assessment

HCMs were seeded in a 96-well plate for 24 h, exposed to 40 mM taurine for 24 h, and treated without glucose and oxygen for 6 h. After the treatment, cell cytotoxicity was determined by the CytoTox 96 Non-radioactive Cytotoxicity Assay Kit (Promega), according to the manufacturer's instructions.

### Measurement of DNA fragmentation

DNA fragmentation, measured by the level of cytosolic mono- and oligonucleosomes, was examined using an ELISA assay (Roche Applied Science). Briefly, homogenates were centrifuged at 15 800 × *g* for 10 min to remove the nuclei. Then, 50 μg of cytosolic fraction was incubated with immunoreagent for 2 h in a streptavidin coated 96-well plate. Next, 100 μl of ABTS solution was applied for color development after washing out the immunoreagent. The extent of DNA fragmentation was assessed in a plate reader at 405 nm.

### Evaluation of apoptosis by flow cytometry

HCMs were washed with PBS twice, centrifuged at 800 × *g* for 3 min and resuspended in ice-cold PBS. The cells were then incubated with FITC-labeled Annexin V-FITC and PI for 30 min at 37 °C by using Annexin V-FITC- FITC apoptosis detection Kit (Dojindo). Excess PI and Annexin V-FITC were then washed off. After that, the cells were fixed and analyzed by flow cytometry using BD Accuri C6 (BD, Sparks, MD, USA) equipped with a 488 nm argon laser light source, with a 525 nm band pass filter for FITC-fluorescence and a 625 nm band pass filter for PI fluorescence. Non-cellular debris and dead cells were gated out, and 30 000 events were collected for each analysis. The unstained cells, the cells stained with Annexin V-FITC alone (no PI) and cells stained with PI alone (no Annexin V-FITC) were used to set up compensation and quadrants. The data are expressed as the percentage of cells with signal above this threshold value as previously described.^35, 36^

### ROS assessment

HCMs were incubated with ROS detection reagents (Life Technologies) and DCF-DA (10 mM) for 60 min at 37 °C in dark, followed by immediately washing with PBS. Fluorescence emission was measured by using a fluorescence microplate (TECAN, infinite F200 PRO, Mannerdorf, Switzerland).

### Measurement of intracellular free Ca^2+^ concentration

Intracellular Ca^**2+**^ levels were determined using the intracellular Ca^**2+**^ probe Fluo-4 AM (Dojindo), which binds Ca^**2+**^ with a 1:1 stoichiometry. CMs were incubated in the dark with Fluo-4 AM for 30 min at 37 °C. Fluorescence emission was measured by using a fluorescence microplate (TECAN, infinite F200 PRO) with an excitation at 494 nm and emission at 516 nm.

### Measurement of ATP content

ATP content was determined with the ATP Bioluminescence Assay Kit HS II (Roche Molecular Biochemicals, South San Francisco, CA, USA), according to the manufacturer's instructions. Briefly, HCMs were washed twice with cold PBS, collected, lysed and mixed with an equal amount of dilution buffer. Lysates were centrifuged at 21100 × *g* for 10 min at 4 °C, and the supernatant was mixed with an equal amount of luciferase reagent. Luminescence was measured by a fluorescence microplate (TECAN, infinite F200 PRO). ATP amounts were calculated from a log–log graph generated for the ATP standard using Magellan software (Rodenberg, Germany). ATP amounts were normalized to protein and presented as percent relative to control. The ATP average for controls was 35 nmol/mg protein, consistent with the reported range. The standard curve linear range was 10^−6^ to 10^−10^ m.

### Measurement of caspase-3 activity

The activity of caspase-3 was assessed by a fluorometric assay, according to the manufacturer's instructions (Biovision, San Francisco, CA, USA). Briefly, 100 μg of protein was incubated on ice for 10 min, followed by the addition of 50 μl of 2 × reaction buffer containing 10 mmol/l DDT. The reaction was initiated by addition of 5 μl of the caspase-specific fluorescent substrate. The mixture was incubated at 37 °C for 2 h in the dark. The activity of caspase-3 was quantified by a fluorescence microplate (TECAN, infinite F200 PRO) with excitation at 400 nm and emission at 505 nm.

### Statistical analysis

The data were expressed as the mean±S.E.M. Comparisons between the means of two groups were performed by unpaired Student's *t*-tests. Multiple groups were analyzed using a one-way ANOVA with a Bonferroni test for *post hoc* analysis. The results were considered statistically significant at *P*<0.05.

## Abbreviations

AMI: acute myocardial infarction
CMs: cardiomyocytes
HCMs: human cardiomyocytes
TauT: taurine transporter
TonE: tonicity-responsive element
TonEBP: tonicity-responsive element-binding protein
CHX: cycloheximide
ChIP: chromatin immunoprecipitation
ROS: reactive oxygen species
LAD: left anterior descending coronary artery ligation
OGD: oxygen glucose deprivation
