# Supplementary figures and images for: TonEBP modulates the protective effect of taurine in ischemia-induced cytotoxicity in cardiomyocytes
Source: Cell Death Dis. 2015 Dec 17;6(12):e2025–. doi: 10.1038/cddis.2015.372 (PMC4720904; doi:10.1038/cddis.2015.372)

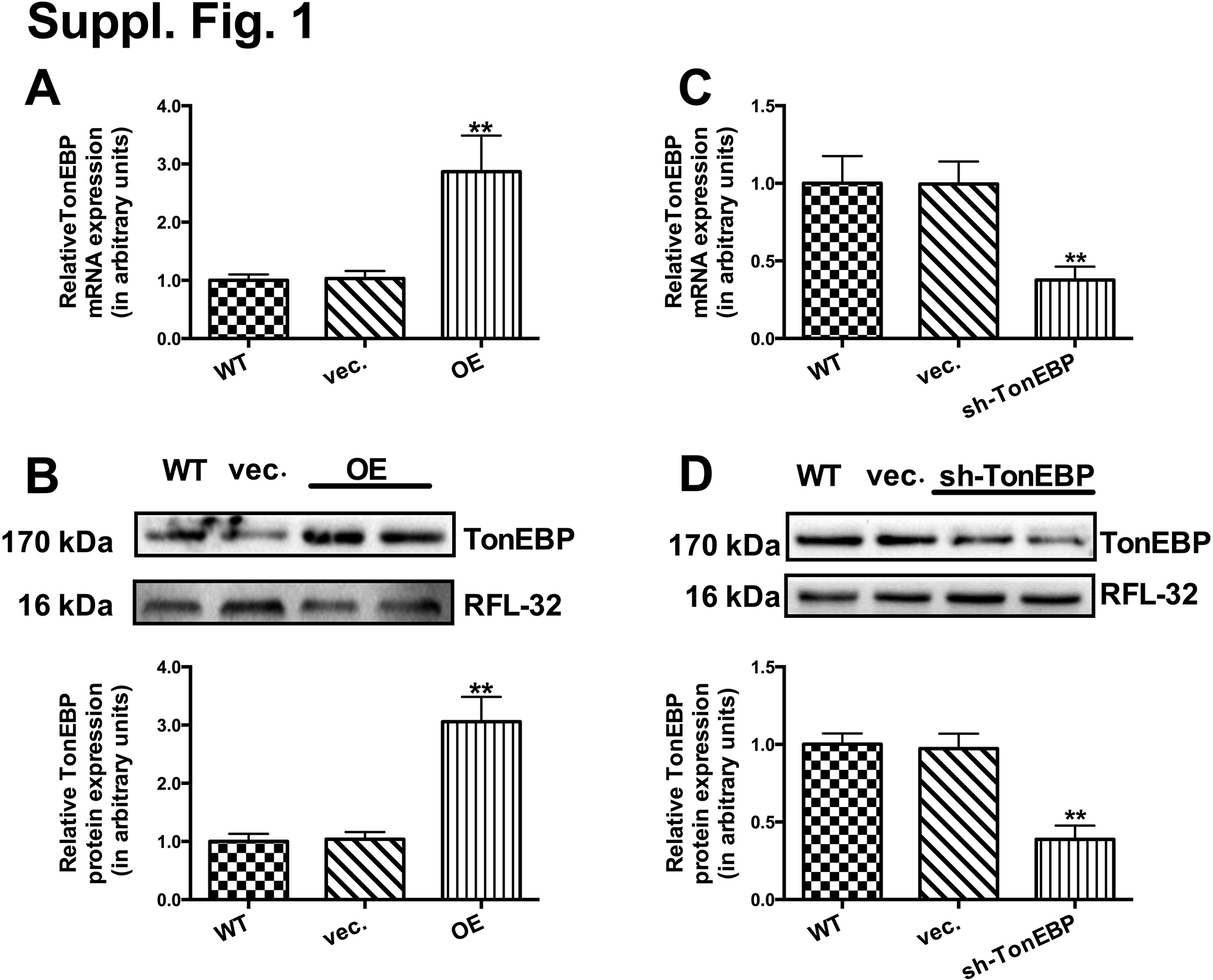

Supplement: Supplementary Figure 1 [file cddis2015372x1.tif]

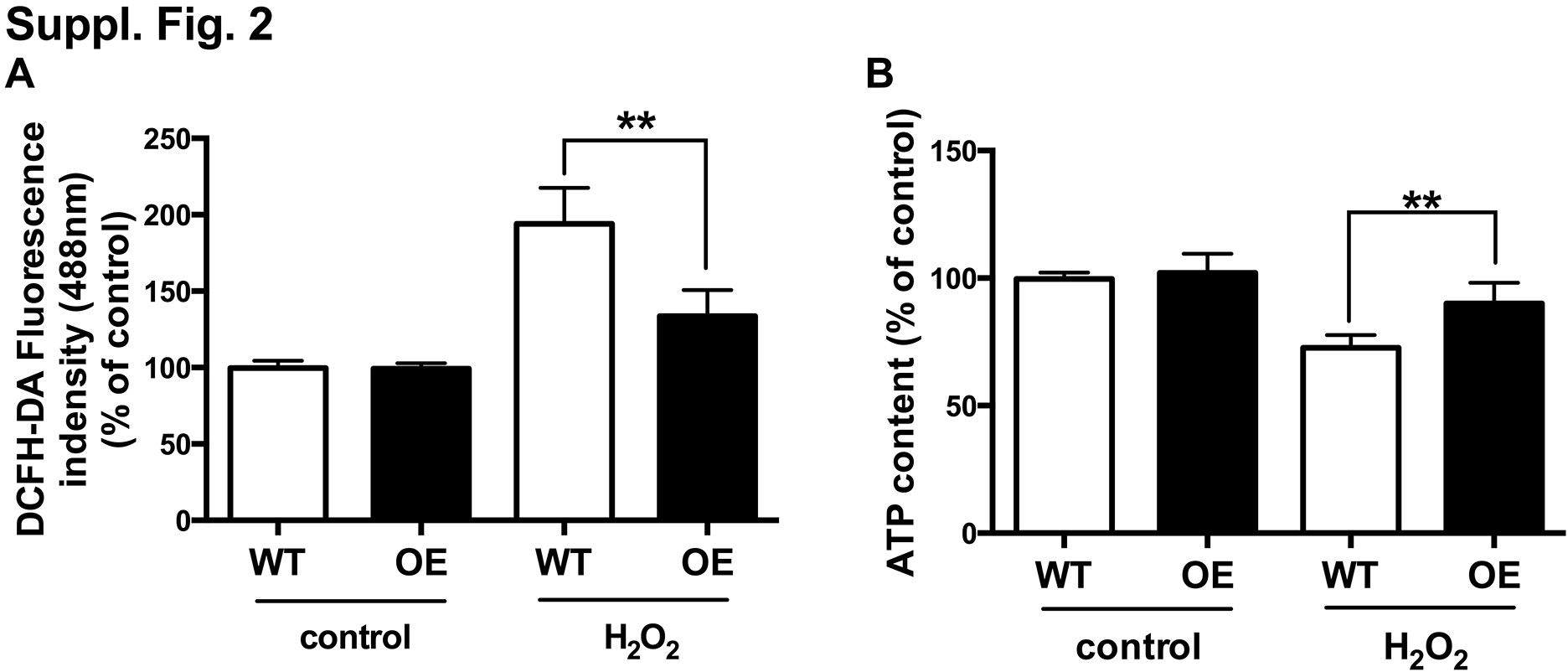

Supplement: Supplementary Figure 2 [file cddis2015372x2.tif]

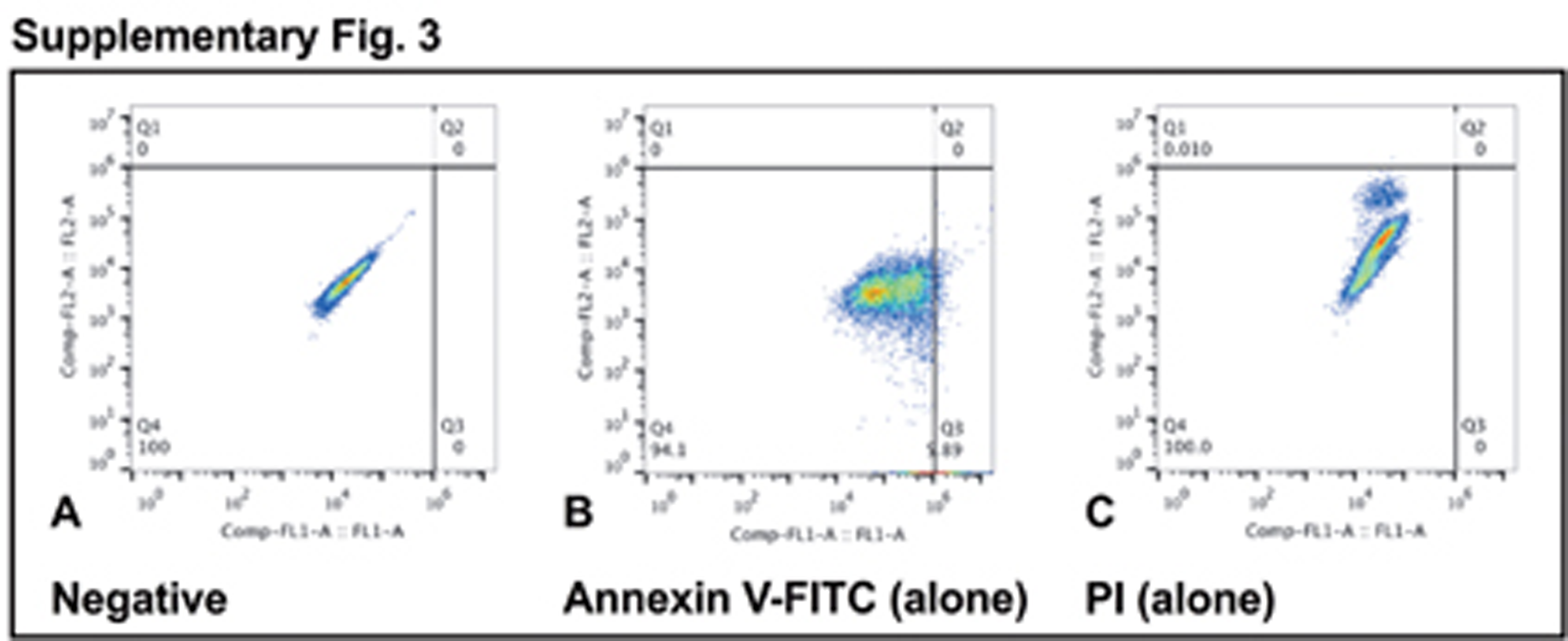

Supplement: Supplementary Figure 3 [file cddis2015372x3.tif]

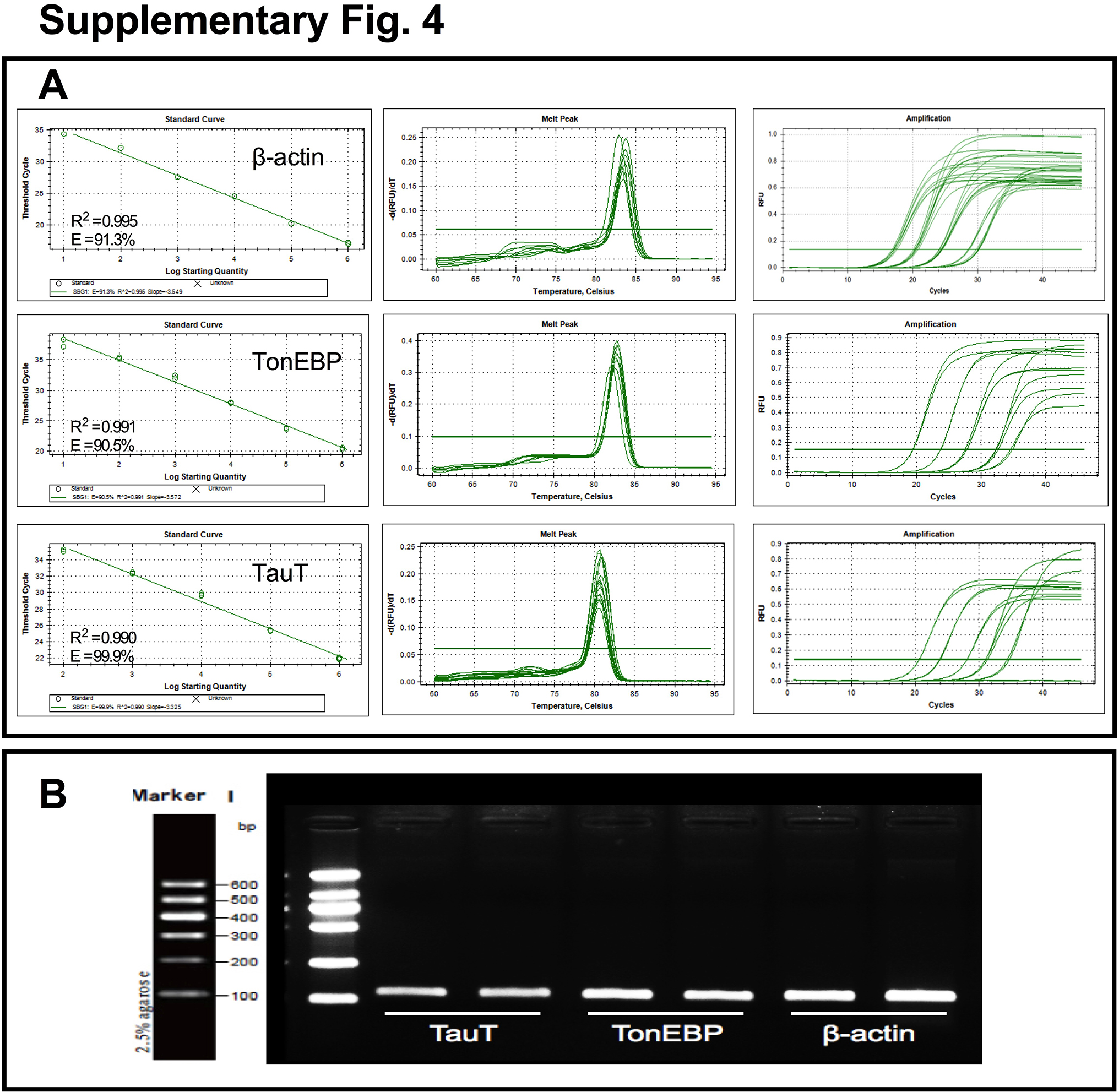

Supplement: Supplementary Figure 4 [file cddis2015372x4.tif]
