# Supplementary material for: TonEBP modulates the protective effect of taurine in ischemia-induced cytotoxicity in cardiomyocytes
Source: Cell Death Dis. 2015 Dec 17;6(12):e2025–. doi: 10.1038/cddis.2015.372 (PMC4720904; doi:10.1038/cddis.2015.372)
Supplement: Supplementary Figure Legends [file cddis2015372x5.doc]

**Supplementary Figure 1. The expression of TonEBP was confirmed by real-time PCR and western blot.** **A and B** confirmed the overexpression of TonEBP; **C and D** confirmed the effects of TonEBP knockdown. (Data were expressed as mean ± SEM. N=6, **, p<0.01; vec. vector; OE, Overexpression)

**Supplementary Figure 2.** The anti-oxidative effects of TonEBP overexpression could also be confirmed in a positive control of hydrogen peroxide. (Data were expressed as mean ± SEM. N=3; **, P<0.01; OE, Overexpression)

**Supplementary Figure 3.** The FACS analysis of **A)** unstained cells, **B)** the cells stained with Annexin V-FITC alone (FL1-A, no PI) and **C)** cells stained with PI alone (FL2-A, no Annexin V-FITC) were performed to set up compensation and quadrants.

**Supplementary Figure 4.** The specificity and efficiency test of all the primers with the thermal program used. **A,** the standard curves, melt peaks and amplification cures in PCR. **B,** the agarose gel electrophoresis of PCR products.
